# Adaptive DCS-SOMP for Localization Parameter Estimation in 5G Networks

**DOI:** 10.3390/s23229073

**Published:** 2023-11-09

**Authors:** Paulo Francisco da Conceição, Flávio Geraldo Coelho Rocha

**Affiliations:** Department of Electrical, Mechanical and Computer Engineering, Federal University of Goiás, Goiânia 74605-010, Brazil; flaviogcr@ufg.br

**Keywords:** 5G, compressed sensing, DCS-SOMP, parameter estimation

## Abstract

In this work, we model a 5G downlink channel using millimeter-wave (mmWave) and massive Multiple-Input Multiple-Output (mMIMO) technologies, considering the following localization parameters: Time of Arrival (TOA), Two-Dimensional Angle of Departure (2D-AoD), and Two-Dimensional Angle of Arrival (2D-AoA), both encompassing azimuth and elevation. Our research focuses on the precise estimation of these parameters within a three-dimensional (3D) environment, which is crucial in Industry 4.0 applications such as smart warehousing. In such scenarios, determining the device localization is paramount, as products must be handled with high precision. To achieve these precise estimations, we employ an adaptive approach built upon the Distributed Compressed Sensing—Subspace Orthogonal Matching Pursuit (DCS-SOMP) algorithm. We obtain better estimations using an adaptive approach that dynamically adapts the sensing matrix during each iteration, effectively constraining the search space. The results demonstrate that our approach outperforms the traditional method in terms of accuracy, speed to convergence, and memory use.

## 1. Introduction

Millimeter-wave (mmWave) and massive Multiple-Input Multiple-Output (mMIMO) technologies are some of the enablers for the future deployment of 5G and beyond 5G networks, constituting essential assets for realizing the full potential of disruptive 5G applications, especially those involving device localization, such as Industry 4.0, Unmanned Aerial Vehicles (UAVs), and Vehicle to Everything (V2X) communications. These technologies significantly enhance the accuracy of localization parameter estimation, such as the Time of Arrival (ToA), Angle of Departure (AoD), and Angle of Arrival (AoA) of signals.

Localization algorithms use localization parameters that can be estimated from the received signal using techniques such as Multiple Signal Classification (MUSIC) [1] and Compressed Sensing (CS) [2]. MUSIC-based methods analyze the cross-correlations among the received signals to identify the angles associated with their peaks in the power spectrum [3]. On the other hand, methods based on CS exploit the sparsity of signals, i.e., instead of acquiring the complete signal, CS enables accurate signal reconstruction using a few important components while the rest are negligible [2]. Among the CS-based methods, we highlight Distributed Compressed Sensing—Subspace Orthogonal Matching Pursuit (DCS-SOMP). DCS-SOMP combines the concept of CS with the Orthogonal Matching Pursuit (OMP) algorithm to handle sparse signals in a distributed environment or in systems with multiple antennas (e.g., mMIMO systems) [4].

In this work, we focus on the DCS-SOMP method, since it aligns more effectively with the sparse nature of mmWave signals. In [5,6], the DCS-SOMP method is applied to parameter estimation in a two-dimensional (2D) environment, providing only a coarse estimation of parameters which is followed by a refinement step using the Space-alternating Generalized Expectation-maximization (SAGE) method [7].

Our proposal has two novelties: first, our approach entails the dynamic adaptation of the sensing matrix, obtaining rapid and accurate parameter estimation through the DCS-SOMP method and second, we perform all parameter estimation within a three-dimensional (3D) environment. Our proposal relies on accurate channel modeling using two-dimensional AoD and AoA parameters coupled with Antennas’s Uniform Circular Arrays (UCA).

The remainder of this article is organized as follows: in Section 2, a literature review is presented. Section 3 outlines the system model, focusing on the channel and received signal modeling. Section 4 elaborates on constructing the sensing matrix and applying the DCS-SOMP method, including the proposed modification for adaptive search in the sensing matrix. In Section 5, simulation results are presented and discussed. Finally, Section 6 concludes the article.

## 2. Related Works

Localization algorithms typically consist of two fundamental steps. First, the estimation of localization parameters extracted from the received signal. Second, using the acquired parameters from the first step, a localization method is employed to determine the position of the mobile station (MS) [5,8,9]. Regarding the first step, we highlight several aspects of the literature.

In [10], the authors estimate AoA and AoD using Sparse Bayes Tensor (SBT) from channel modeling using MIMO and mmWave. However, due to the use of a linear array of antennas, the proposed approach can only determine the azimuth of AoA and AoD and does not enable ToA estimation. In [8], the authors conduct indoor localization using channel modeling and ray tracing. They use two Uniform Linear Arrays (ULA), one perpendicular to the other, to extract the elevation angle. However, the method does not estimate AoD, thus preventing the use of algorithms dependent on this parameter.

In [5], the authors propose a comprehensive localization algorithm using MIMO, mmWave, and ULA. They employ the DCS-SOMP method for parameter estimation. Due to the linear antenna array, the method applies to a 2D environment. Additionally, the DCS-SOMP method provides only a coarse parameter estimate, demanding further fine-tuning using the SAGE method. In [11], the authors propose a 2D-AoA and 2D-AoD estimation using the MUSIC algorithm. The method utilizes a channel model with a rectangular Uniform Rectangular Array (URA). However, their proposal does not estimate ToA and only allows coarse angle estimation. Similarly to [5], the proposal in [11] requires fine-tuning techniques to provide accurate parameter estimations. In [12], the authors propose a technique named spatial spectrum fusion estimation and localization (SSFEAL) for performing 2D-AoA estimation using UCA in a MIMO mmWave channel.

In this work, we go beyond the related studies by proposing a joint estimation method of ToA, 2D-AoD, and 2D-AoA, enabling the use of these parameters in 3D localization algorithms in multipath environments. Furthermore, our proposed method accurately estimates the parameters without additional fine-tuning techniques. We employ a MIMO mmWave channel model with UCA to determine 2D-AoD and 2D-AoA, consistent with the works in [12,13]. Table 1 compares our proposal with others discussed in this section.

Table 1 demonstrates aspects of some related works and our proposal. Column 2 indicates the method used for parameter estimation. Column 3 displays the antenna array, while columns 4 to 6 specify whether the method estimates ToA, 2D-AoD, and 2D-AoA parameters, respectively. Our proposal presents the advantage of estimating all considered localization parameters (ToA, 2D-AoD, and 2D-AoA), while other methods estimate only some of them, or consider only two-dimensional (2D) environments. Additionally, our proposal utilizes the DCS-SOMP itself to achieve high accuracy, while other methods rely on extra algorithms for a refinement step. Although estimating five parameters increases the number of elements in the sensing matrix, our adaptive approach reduces execution time and yields more accurate results.

## 3. System Model

As outlined in the 3rd Generation Partnership Project (3GPP) guidelines released in the TR 38.901 V17.0.0 [14], we analyze a MIMO system with $N_{t}$ transmitter antennas at the base station (BS) and $N_{r}$ receiver antennas at the MS. This system operates at carrier frequency $f_{c}$ and with bandwidth *B*. Similarly to [5], we consider the BS to send *G* signals to the receiver, where the *g*-th signal is composed of $M_{t}$ symbols. Furthermore, we consider an indoor environment where there are *L* paths for the signals being transmitted from a single BS to the MS, such paths can be classified as Line of Sight (LoS) and single-bounce Non-Line of Sight (NLoS) paths. Multiple-bounce paths are not taken into account due to their limited reception strength at mmWave frequencies [15]. Additionally, we assume perfect clock synchronization, as in [16], enabling accurate ToA estimation.

**Figure 1:**
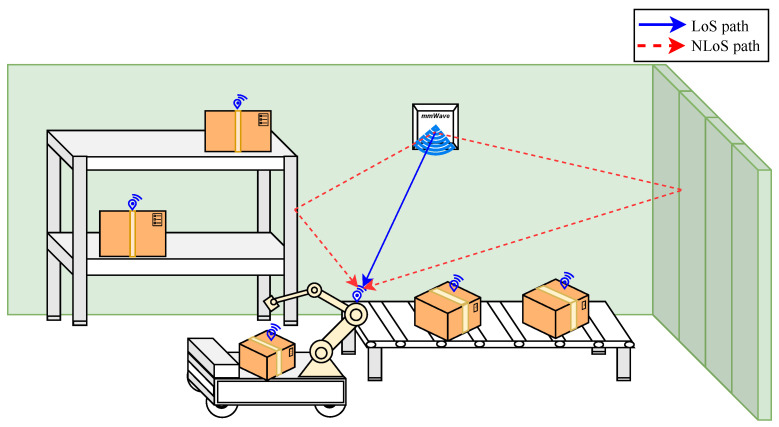
Indoor Localization Scenario with LoS and NLoS Paths: in a smart warehouse, a forklift is connected to a mmWave 5G network for product transport.

Figure 1 illustrates a typical Industry 4.0 scenario in a smart warehouse where a forklift moves products from the conveyor belt to the shelves. In such a scenario, the localization and tracking of the forklift can be achieved using geometry-based methods, which rely on accurate estimation of localization parameters [5,17].

As in [14], we assume the channel’s dependency on the response vector. Thus, for the *n*-th subcarrier (where n=0,...,N−1), the channel matrix H can be represented as follows:

(1) $$H\left[n\right]=\sum_{l=1}^{L}\rho_{l}h_{l}e^{\frac{-j2\pi \left(n-1\right)\tau_{l}}{NT_{s}}}\sqrt{N_{t}}a_{t}^{H}\left(\phi_{l}^{az},\phi_{l}^{el}\right)\sqrt{N_{r}}a_{r}\left(\theta_{l}^{az},\theta_{l}^{el}\right),$$

where, for the *l*-th path, $\rho_{l}$ represents the pathloss, $h_{l}$ stands for the complex channel gain, $\tau_{l}$ is the ToA, $\phi_{l}^{az}$ is the azimuth AoD, $\phi_{l}^{el}$ is the elevation AoD, $\theta_{l}^{az}$ is the azimuth AoA, $\theta_{l}^{el}$ is the elevation AoA, and $T_{s}=1/B$ denotes the sampling period. In order to take into account 2D-AoD, i.e., ($\phi_{l}^{az}$, $\phi_{l}^{el}$) and 2D-AoA, i.e., ($\theta_{l}^{az}$, $\theta_{l}^{el}$), we employ a Uniform Circular Array (UCA) as in [13], thus we define the response vectors $a_{t}$ and $a_{r}$, as follows:

(2) $$a_{t}\left(\phi_{l}^{az},\phi_{l}^{el}\right)=\left[1,e^{j\frac{2\pi }{\lambda }rsin\left(\phi_{l}^{el}\right)cos\left(\phi_{l}^{az}-sin\left(1\right)\right)},\ldots ,e^{j\frac{2\pi }{\lambda }rsin\left(\phi_{l}^{el}\right)cos\left(\phi_{l}^{az}-sin\left(N_{t}-1\right)\right)}\right],$$

(3) $$a_{r}\left(\theta_{l}^{az},\theta_{l}^{el}\right)=\left[1,e^{j\frac{2\pi }{\lambda }rsin\left(\theta_{l}^{el}\right)cos\left(\theta_{l}^{az}-sin\left(1\right)\right)},\ldots ,e^{j\frac{2\pi }{\lambda }rsin\left(\theta_{l}^{el}\right)cos\left(\theta_{l}^{az}-sin\left(N_{r}-1\right)\right)}\right],$$

where λ is the wavelength, and *r* is the radius of the UCA. When a massive antenna array is used, as described in [13], the radius can be defined as $r=\left(N_{t}-1\right)\frac{\lambda /2}{2\pi }$ for $a_{t}\left(\phi_{l}^{az},\phi_{l}^{el}\right)$, and as $r=\left(N_{r}-1\right)\frac{\lambda /2}{2\pi }$ for $a_{r}\left(\theta_{l}^{az},\theta_{l}^{el}\right)$.

Finally, the received signal for subcarrier *n* and transmission *g* can be expressed as follows:

(4) $$y^{\left(g\right)}\left[n\right]=H\left[n\right]x^{\left(g\right)}\left[n\right]+w\left[n\right],$$

where $x^{\left(g\right)}$ represents the signal with the transmitted data symbols, and w denotes a Gaussian noise vector with a zero mean and variance $\sigma^{2}$.

## 4. Proposed Method

For ease of understanding, we divide our proposal into two stages: (1) sensing matrix construction and (2) 3D parameter estimation using the DCS-SOMP algorithm.

### 4.1. Sensing Matrix Construction

According to [14], the azimuth angle belongs to the interval [0,2π] and the elevation angle belongs to the interval [0,π]. Therefore, we have $q_{az}$ candidates for azimuth angles uniformly spaced within the interval [0,2π], and $q_{el}$ candidates for elevation angles uniformly spaced within the interval [0,π]. We define ${\tilde{\phi }}^{\left(0\right)}=\left[{\tilde{\phi }}_{1}^{\left(0\right)},\ldots ,{\tilde{\phi }}_{q_{az}}^{\left(0\right)}\right]$, ${\dot{\phi }}^{\left(0\right)}=\left[{\dot{\phi }}_{1}^{\left(0\right)},\ldots ,{\dot{\phi }}_{q_{el}}^{\left(0\right)}\right]$ as candidates for AoD azimuth and elevation, respectively, and ${\tilde{\theta }}^{\left(0\right)}=\left[{\tilde{\theta }}_{1}^{\left(0\right)},\ldots ,{\tilde{\theta }}_{q_{az}}^{\left(0\right)}\right]$, ${\dot{\theta }}^{\left(0\right)}=\left[{\dot{\theta }}_{1}^{\left(0\right)},\ldots ,{\dot{\theta }}_{q_{el}}^{\left(0\right)}\right]$ as candidates for AoA azimuth and elevation, respectively. We introduce the matrix $U_{t}^{\left(0\right)}$ of dimensions $N_{t}\times q_{az}q_{el}$ containing the response vectors for each possible combination of azimuth and elevation for AoD:

(5) $$U_{t}^{\left(0\right)}=\left[a_{t}\left({\tilde{\phi }}^{\left(0\right)},{\dot{\phi }}^{0}\right)\right].$$

Similarly, we create the matrix $U_{r}^{\left(0\right)}$ of dimensions $N_{r}\times q_{az}q_{el}$ containing the response vectors for each possible combination of azimuth and elevation for AoA:

(6) $$U_{r}^{\left(0\right)}=\left[a_{t}\left({\tilde{\theta }}^{\left(0\right)},{\dot{\theta }}^{\left(0\right)}\right)\right].$$

Finally, we obtain the sensing matrix $\omega^{\left(0\right)}$ as follows:

(7) $$\omega^{\left(0\right)}\left[n\right]={\left(U_{t}^{\left(0\right)}x^{\left(g\right)}\left[n\right]\right)}^{T}⊗U_{r}^{\left(0\right)},$$

where $x^{\left(g\right)}$ represents the transmitted data and ⊗ denotes the Kronecker product. The sensing matrix ω has dimensions of $\left(N_{t}M_{t}\right)\times {\left(q_{az}q_{el}\right)}^{2}$. As a result, the number of elements in ω is directly related and highly sensitive to the values of $q_{az}$ and $q_{el}$.

**Figure 2:**
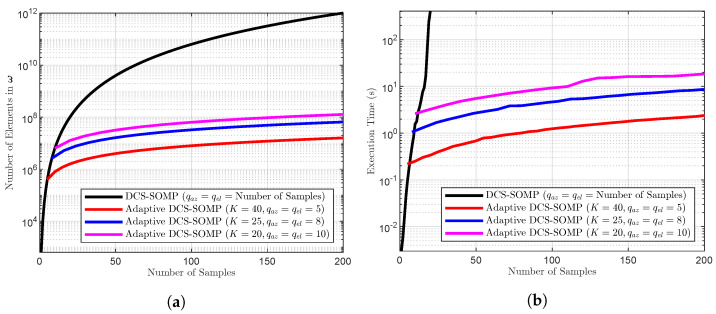
Comparison between DCS-SOMP and Adaptive DCS-SOMP: (**a**) Variation in Sensing Matrix Size (Number of Elements) and (**b**) Execution Time, both regarding the number of samples.

In Section 5, we have demonstrated that increasing the number of candidates ($q_{az}$, $q_{el}$) for DCS-SOMP is crucial to achieving accurate estimations of 2D-AoD and 2D-AoA. However, an increased number of candidates also leads to increased memory usage, as shown in Figure 2. Our proposed modification to DCS-SOMP introduces an adaptive search approach, dynamically adjusting the sensing matrix during each iteration, as detailed in Section 4.3, to address this issue.

### 4.2. DCS-SOMP Approach for 3D Parameter Estimation

The DCS-SOMP method determines the index ${\tilde{h}}_{l}$ of the maximum correlation between (4) and (7). Therefore, for the *l*-th path, ${\tilde{h}}_{l}$ is defined as follows:

(8) $${\tilde{h}}_{l}=\underset{m=1,\ldots ,{\left(q_{az}q_{el}\right)}^{2}}{\mathrm{argmax}}\sum_{n=1}^{N}\frac{|\omega_{m}^{H}\left[n\right]R_{l}\left[n\right]|}{∥\omega_{m}{\left[n\right]∥}_{2}},$$

where $R_{l}$ represents the received signal’s residue and *m* represents each column in ω. $R_{l}=y$ when l=1.

The index ${\tilde{h}}_{l}$ is converted into a combination of $\left(\phi_{l}^{az},\phi_{l}^{el}\right)$ and $\left(\theta_{l}^{az},\theta_{l}^{el}\right)$. The 2D-AoD $\left(\phi_{l}^{az},\phi_{l}^{el}\right)$ is determined as follows:

(9) $$\begin{array}{ccc} I_{\phi }^{l}=\frac{{\tilde{h}}_{l}}{q_{az}q_{el}}, & \iota_{l}^{az}=\left\lfloor \frac{I_{\phi }^{l}}{q_{az}}\right\rfloor , & \iota_{l}^{el}=I_{\phi }^{l}\mathrm{mod}q_{el}, \end{array}$$

(10) $$\begin{array}{cc} \phi_{l}^{az}={\tilde{\phi }}_{\iota_{l}^{az}}^{\left(0\right)}, & \phi_{l}^{el}={\dot{\phi }}_{\iota_{l}^{el}}^{\left(0\right)}. \end{array}$$

Similarly, the 2D-AoA $\left(\theta_{l}^{az},\theta_{l}^{el}\right)$ is determined as follows:

(11) $$\begin{array}{ccc} I_{\theta }^{l}={\tilde{h}}_{l}-I_{\phi }^{l}q_{az}q_{el}, & \gamma_{l}^{az}=\left\lfloor \frac{I_{\theta }^{l}}{q_{az}}\right\rfloor , & \gamma_{l}^{el}=I_{\theta }^{l}\mathrm{mod}q_{el}, \end{array}$$

(12) $$\begin{array}{cc} \theta_{l}^{az}={\tilde{\theta }}_{\gamma_{l}^{az}}^{\left(0\right)}, & \theta_{l}^{el}={\dot{\theta }}_{\gamma_{l}^{el}}^{\left(0\right)}. \end{array}$$

Finally, the DCS-SOMP method updates the residual for the *n*-th subcarrier as follows:

(13) $$\beta_{l}\left[n\right]=\frac{\omega_{{\tilde{h}}_{l}}^{H}\left[n\right]R_{l}\left[n\right]}{∥\omega_{{\tilde{h}}_{l}}{\left[n\right]∥}_{2}^{2}},$$

(14) $$R_{l+1}\left[n\right]=R_{l}\left[n\right]-\beta_{l}\left[n\right]\omega_{{\tilde{h}}_{l}}\left[n\right].$$

Similar to [5], the estimation of ToA provides a maximum distance of $NT_{s}c$ (m). Therefore, ToA can be estimated as follows:

(15) $$\mathrm{distance}=\frac{1}{N}\left[\sum_{n=1}^{N}\beta_{l}\left[n\right]\right]NT_{s}\frac{c}{2\pi },$$

(16) $$\tau_{l}=\left\{\begin{array}{c} \mathrm{distance}/c\;,\;\mathrm{if}\;\mathrm{distance}\;\geq 0\\ (\mathrm{distance}+NT_{s}c)/c\;,\;\mathrm{if}\;\mathrm{distance}<0, \end{array}\right.$$

where *c* is the speed of light.

### 4.3. Adaptive Search in DCS-SOMP Approach for 3D Parameter Estimation

**Figure 3:**
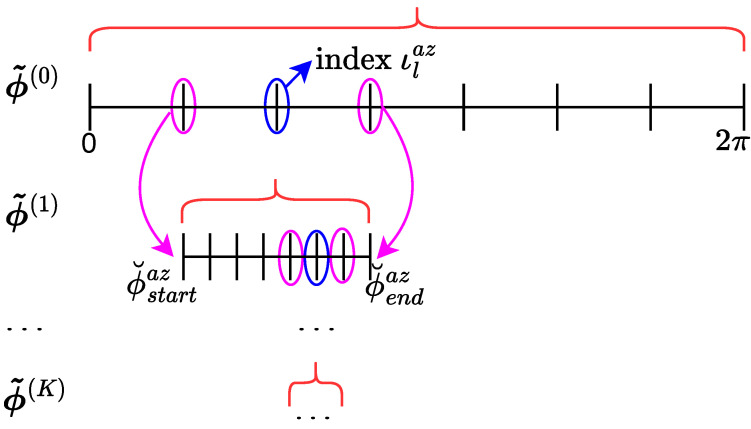
Adaptive Procedure for Selecting Eligible Candidates for Azimuth AoD.

We introduce an adaptive approach to enhance the parameters estimated in the two aforementioned stages: (1) sensing matrix construction and (2) application of the DCS-SOMP approach for 3D parameter estimation. Our method dynamically adapts the search space for each angle to be estimated, i.e., 2D-AoD and 2D-AoA. To illustrate how dynamic adaptation is performed, Figure 3 demonstrates the improvements made in each iteration using our adaptive approach for the azimuth AoD angle. In summary, in iteration *k*, the search interval (${\tilde{\phi }}^{\left(k\right)}$) is dynamically adapted using the index ($\iota_{l}^{az}$) of the previous search interval (${\tilde{\phi }}^{\left(k-1\right)}$). New parameters are estimated in each iteration. The process continues until a predetermined number of iterations (*K*) is reached or a pre-established threshold (ϱ) is exceeded.

We adaptively calculate the candidates for AoD azimuth angle (${\tilde{\phi }}^{\left(k\right)}$) with $q_{az}$ values that are uniformly spaced within a new interval $\left[{\overset{˘}{\phi }}_{start}^{az},{\overset{˘}{\phi }}_{end}^{az}\right]$, where

(17) $$\begin{array}{cc} {\overset{˘}{\phi }}_{start}^{az}=\left\{\begin{array}{c} {\tilde{\phi }}_{\iota_{l}^{az}-1}^{\left(k-1\right)},\;\mathrm{if}\;\iota_{l}^{az}>1\\ 0,\;\mathrm{if}\;\iota_{l}^{az}=1 \end{array}\right.\;\mathrm{and}\;\; & {\overset{˘}{\phi }}_{end}^{az}=\left\{\begin{array}{c} {\tilde{\phi }}_{\iota_{l}^{az}+1}^{\left(k-1\right)},\;\mathrm{if}\;\iota_{l}^{az}<q_{az}\\ q_{az},\;\mathrm{if}\;\iota_{l}^{az}=q_{az} \end{array}\right. \end{array}.$$

Similarly, we adaptively calculate the candidates for AoD elevation angle (${\dot{\phi }}^{\left(k\right)}$) with $q_{el}$ values that are uniformly spaced within a new interval $\left[{\overset{˘}{\phi }}_{start}^{el},{\overset{˘}{\phi }}_{end}^{el}\right]$, where

(18) $$\begin{array}{cc} {\overset{˘}{\phi }}_{start}^{el}=\left\{\begin{array}{c} {\dot{\phi }}_{\iota_{l}^{el}-1}^{\left(k-1\right)},\;\mathrm{if}\;\iota_{l}^{el}>1\\ 0,\;\mathrm{if}\;\iota_{l}^{el}=1 \end{array}\right.\;\mathrm{and}\;\; & {\overset{˘}{\phi }}_{end}^{el}=\left\{\begin{array}{c} {\dot{\phi }}_{\iota_{l}^{el}+1}^{\left(k-1\right)},\;\mathrm{if}\;\iota_{l}^{el}<q_{el}\\ q_{el},\;\mathrm{if}\;\iota_{l}^{el}=q_{el} \end{array}\right. \end{array}.$$

For the AoA azimuth angle, we adaptively calculate the candidates (${\tilde{\theta }}^{\left(k\right)}$) with $q_{az}$ values that are uniformly spaced within a new interval $\left[{\overset{˘}{\theta }}_{start}^{az},{\overset{˘}{\theta }}_{end}^{az}\right]$, where

(19) $$\begin{array}{cc} {\overset{˘}{\theta }}_{start}^{az}=\left\{\begin{array}{c} {\tilde{\theta }}_{\gamma_{l}^{az}-1}^{\left(k-1\right)},\;\mathrm{if}\;\gamma_{l}^{az}>1\\ 0,\;\mathrm{if}\;\gamma_{l}^{az}=1 \end{array}\right.\;\mathrm{and}\;\; & {\overset{˘}{\theta }}_{end}^{az}=\left\{\begin{array}{c} {\tilde{\theta }}_{\gamma_{l}^{az}+1}^{\left(k-1\right)},\;\mathrm{if}\;\gamma_{l}^{az}<q_{az}\\ q_{az},\;\mathrm{if}\;\gamma_{l}^{az}=q_{az} \end{array}\right. \end{array}.$$

Similarly, we adaptively calculate the candidates for AoA elevation angle (${\dot{\theta }}^{\left(k\right)}$) with $q_{el}$ values that are uniformly spaced within a new interval $\left[{\overset{˘}{\theta }}_{start}^{el},{\overset{˘}{\theta }}_{end}^{el}\right]$, where

(20) $$\begin{array}{cc} {\overset{˘}{\theta }}_{start}^{el}=\left\{\begin{array}{c} {\dot{\theta }}_{\gamma_{l}^{el}-1}^{\left(k-1\right)},\;\mathrm{if}\;\gamma_{l}^{el}>1\\ 0,\;\mathrm{if}\;\gamma_{l}^{el}=1 \end{array}\right.\;\mathrm{and}\;\; & {\overset{˘}{\theta }}_{end}^{el}=\left\{\begin{array}{c} {\dot{\theta }}_{\gamma_{l}^{el}+1}^{\left(k-1\right)},\;\mathrm{if}\;\gamma_{l}^{el}<q_{el}\\ q_{el},\;\mathrm{if}\;\gamma_{l}^{el}=q_{el} \end{array}\right. \end{array}.$$

Thus, using ${\tilde{\phi }}^{\left(k\right)}$, ${\dot{\phi }}^{\left(k\right)}$, ${\tilde{\theta }}^{\left(k\right)}$, and ${\dot{\theta }}^{\left(k\right)}$ we determine the new sensing matrix as follows:

(21) $$U_{t}^{\left(k\right)}=\left[a_{t}\left({\tilde{\phi }}^{\left(k\right)},{\dot{\phi }}^{k}\right)\right],$$

(22) $$U_{r}^{\left(k\right)}=\left[a_{t}\left({\tilde{\theta }}^{\left(k\right)},{\dot{\theta }}^{\left(k\right)}\right)\right],$$

(23) $$\omega^{\left(k\right)}\left[n\right]={\left(U_{t}^{\left(k\right)}x^{\left(g\right)}\left[n\right]\right)}^{T}⊗U_{r}^{\left(k\right)}.$$

Using $R_{l}$ and $\omega^{\left(k\right)}$, we determine new values for ($\phi_{l}^{az}$, $\phi_{l}^{el}$), ($\theta_{l}^{az}$, $\theta_{l}^{el}$), and $\tau_{l}^{el}$ from (10), (12), and (16), respectively, which initiates a new iteration. Although the adaptive step enhances the sensing matrix for 2D-AoD and 2D-AoA estimation, it also yields improvements in ToA estimation. The continual improvement of the maximum correlation with each iteration in the adaptive process positively impacts the ToA estimation accuracy.

Therefore, our adaptive approach involves using a smaller number for $q_{az}$ and $q_{el}$, enabling us to achieve a high number of samples in the overall search space while consuming less memory. This is due to the reduced size of the matrix ω in terms of its total number of elements. Additionally, our approach requires less time than the simple method while maintaining greater accuracy.

Figure 2a,b clearly demonstrates that when using the DCS-SOMP method, a small sample space is accommodated, should memory space or execution time be constrained. The simulations reveal that when using over 18 samples in the sensing matrix, the DCS-SOMP method becomes impractical in terms of execution time and memory. On the other hand, the adaptive method consumes less memory, exhibits shorter execution times, and is capable of accommodating larger sample spaces. Furthermore, the sample space accommodated by the adaptive method consistently remains close to the actual value, enhancing its precision. We calculate the number of elements ($q_{\omega }$) in ω using the formula: $q_{\omega }=\left(\left(N_{t}M_{t}\right){\left(q_{az}q_{el}\right)}^{2}\right)K$, where K=1 for DCS-SOMP. To generate Figure 2a, we arbitrarily set the number of antennas at the transmitter to $N_{t}=32$ and the number of transmitted symbols to $M_{t}=20$, similar results were obtained with other values.

Algorithm 1 outlines our proposal. The adaptive search step is called at line 17. Algorithm 2 provides a summary of the adaptive search step.
**Algorithm 1** Modified DCS-SOMP**Input:** y, $\omega^{\left(0\right)}$, ${\tilde{\phi }}^{\left(0\right)}$, ${\dot{\phi }}^{\left(0\right)}$, ${\tilde{\theta }}^{\left(0\right)}$, ${\dot{\theta }}^{\left(0\right)}$, $q_{az}$, $q_{el}$, *K*, *L*, *N***Output:** $\phi_{l}^{az}$, $\phi_{l}^{el}$, $\theta_{l}^{az}$, $\theta_{l}^{el}$, $\tau_{l}$ 1:$R_{1}\leftarrow y$ 2:**for** l←1 to *L* **do** 3:    ${\tilde{h}}_{l}\leftarrow -1$ 4:    $\max_{corr}\leftarrow -1$                                 ▹ *Maximum Correlation* 5:    **for** m←1 to ${\left(q_{az}q_{el}\right)}^{2}$ **do** 6:        corr←0 7:        **for** n←1 to *N* **do** 8:           $\mathrm{corr}\leftarrow \mathrm{corr}+\frac{{\left[\omega_{m}^{\left(0\right)}\left[n\right]\right]}^{T}R_{l}\left[n\right]}{∥\omega_{m}^{\left(0\right)}{\left[n\right]∥}_{2}}$ 9:        **end for**10:        **if** $\mathrm{corr}>\max_{corr}$ **then**11:           ${\tilde{h}}_{l}\leftarrow m$12:        **end if**13:    **end for**14:    Determine $\iota_{l}^{az}$, $\iota_{l}^{el}$, $\gamma_{l}^{az}$ and $\gamma_{l}^{el}$ from (9) and (11)15:    Determine ($\phi_{l}^{az}$, $\phi_{l}^{el}$) from (10)16:    Determine ($\theta_{l}^{az}$, $\theta_{l}^{el}$) from (12)17:    Using [$R_{l}$, $\iota_{l}^{az}$, $\iota_{l}^{el}$, $\gamma_{l}^{az}$, $\gamma_{l}^{el}$, ${\tilde{\phi }}^{\left(0\right)}$, ${\dot{\phi }}^{\left(0\right)}$, ${\tilde{\theta }}^{\left(0\right)}$, *K*, *N*, $q_{az}$, $q_{el}$] as input, fine-tune [$\phi_{l}^{az}$,$\phi_{l}^{el}$,$\theta_{l}^{az}$,$\theta_{l}^{az}$, $\tau_{l}$] using Algorithm 218:    **for** n←1 to *N* **do**19:        Determine $\beta_{l}\left[n\right]$ from (13)20:        Determine $R_{l+1}\left[n\right]$ from (14)21:    **end for**22:    Determine $\tau_{l}$ from (16)                    ▹ *Execute solely if the adaptive step is not called*23:**end for**

**Algorithm 2** Adaptive Search**Input:** $R_{l}$, $\iota_{l}^{az}$, $\iota_{l}^{el}$, $\gamma_{l}^{az}$, $\gamma_{l}^{el}$, ${\tilde{\phi }}^{\left(0\right)}$, ${\dot{\phi }}^{\left(0\right)}$, ${\tilde{\theta }}^{\left(0\right)}$, *K*, *N*, $q_{az}$, $q_{el}$**Output:** $\phi_{l}^{az}$, $\phi_{l}^{el}$, $\theta_{l}^{az}$, $\theta_{l}^{el}$, $\tau_{l}$
 1:**for** k←1 to *K* **do** 2:    Determine $\left[{\overset{˘}{\phi }}_{start}^{az},{\overset{˘}{\phi }}_{end}^{az}\right]$ from (17) 3:    Determine $\left[{\overset{˘}{\phi }}_{start}^{el},{\overset{˘}{\phi }}_{end}^{el}\right]$ from (18) 4:    Determine $\left[{\overset{˘}{\theta }}_{start}^{az},{\overset{˘}{\theta }}_{end}^{az}\right]$ from (19) 5:    Determine $\left[{\overset{˘}{\theta }}_{start}^{el},{\overset{˘}{\theta }}_{end}^{el}\right]$ from (20) 6:    ${\tilde{\phi }}^{\left(k\right)}\leftarrow \left[{\overset{˘}{\phi }}_{start}^{az},\ldots ,{\overset{˘}{\phi }}_{end}^{az}\right]$           ▹$q_{az}$ *values uniformly spaced* 7:    ${\dot{\phi }}^{\left(k\right)}\leftarrow \left[{\overset{˘}{\phi }}_{start}^{el},\ldots ,{\overset{˘}{\phi }}_{end}^{el}\right]$           ▹$q_{el}$ *values uniformly spaced* 8:    ${\tilde{\theta }}^{\left(k\right)}\leftarrow \left[{\overset{˘}{\theta }}_{start}^{az},\ldots ,{\overset{˘}{\theta }}_{end}^{az}\right]$            ▹$q_{az}$ *values uniformly spaced* 9:    ${\tilde{\theta }}^{\left(k\right)}\leftarrow \left[{\overset{˘}{\theta }}_{start}^{el},\ldots ,{\overset{˘}{\theta }}_{end}^{el}\right]$            ▹$q_{el}$ *values uniformly spaced*10:    **for** n←1 to *N* **do**11:        Determine $\omega^{\left(k\right)}\left[n\right]$ from (23)12:    **end for**13:    ${\tilde{h}}_{l}\leftarrow -1$14:    $\max_{corr}\leftarrow -1$                  ▹ *Maximum Correlation*15:    **for** m←1 to ${\left(q_{az}q_{el}\right)}^{2}$ **do**16:        corr←017:        **for** n←1 to *N* **do**18:           $\mathrm{corr}\leftarrow \mathrm{corr}+\frac{{\left[\omega_{m}^{\left(k\right)}\left[n\right]\right]}^{T}R_{l}\left[n\right]}{∥\omega_{m}^{\left(k\right)}{\left[n\right]∥}_{2}}$19:        **end for**20:        **if** $\mathrm{corr}>\max_{corr}$ **then**21:           ${\tilde{h}}_{l}\leftarrow m$22:        **end if**23:    **end for**24:    Determine $\iota_{l}^{az}$, $\iota_{l}^{el}$, $\gamma_{l}^{az}$ and $\gamma_{l}^{el}$ from (9) and (11)25:    Determine ($\phi_{l}^{az}$, $\phi_{l}^{el}$) from (10)26:    Determine ($\theta_{l}^{az}$, $\theta_{l}^{el}$) from (12)27:    **for** n←1 to *N* **do**28:        Determine $\beta_{l}\left[n\right]$ from (13)29:    **end for**30:    Determine $\tau_{l}$ from (16)31:
**end for**



## 5. Results

In this article, we conducted simulations using ${\mathrm{MATLAB}}^{®}$ software version R2012b (used under an academic license) installed in a computer running Windows 11 as the operating system and with the following hardware configurations: 2.5 GHz Intel Core i5-10300H processor, 16 GB RAM, and NVIDIA GTX 1650 as the dedicated video card.

We selected arbitrary actual values for two paths as described in Table 2. We set $q_{az}=18$ and $q_{el}=18$ when not using the adaptive search, i.e., DCS-SOMP, as this is the maximum value our simulator could process. When using the adaptive search, we set $q_{az}=6$ and $q_{el}=6$. Additionally, we defined the parameters as follows: $f_{c}=28$ GHz, B=100 MHz, $N_{t}=64$, $N_{r}=64$, N=10, and $M_{t}=20$. To establish a maximum number of iterations, we determined the difference between the estimated value in iteration *k* and the estimated value in iteration k−1, and as long as this difference is greater than the threshold ($\varrho =10^{-6}$), the next iteration will be performed.

**Figure 4:**
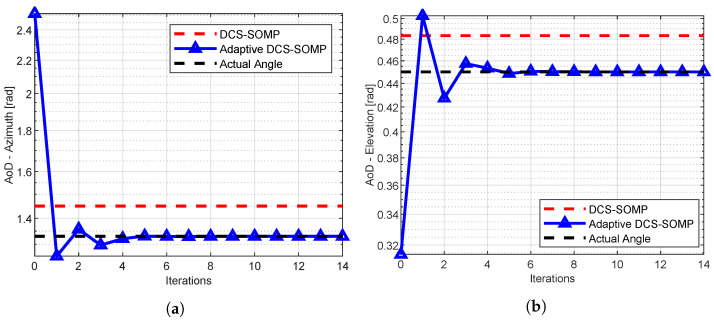
Comparison of (**a**) Azimuth AoD estimation and (**b**) Elevation AoD estimation results using DCS-SOMP and adaptive DCS-SOMP. The outcomes pertain to Path 1. In the DCS-SOMP case, $q_{az}$ and $q_{el}$ are both set to 18, whereas in the adaptive DCS-SOMP scenario, $q_{az}$ and $q_{el}$ are both set to 6.

**Figure 5:**
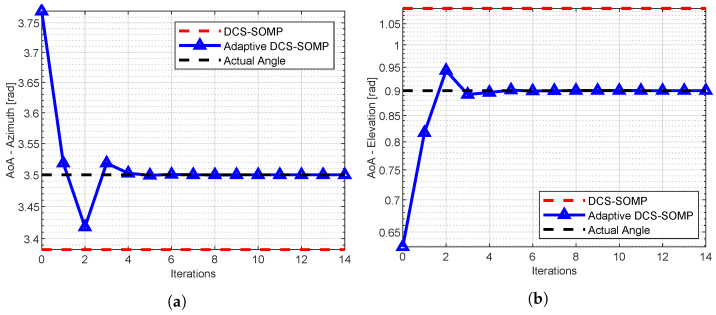
Comparison of (**a**) Azimuth AoA estimation and (**b**) Elevation AoA estimation results using DCS-SOMP and adaptive DCS-SOMP. The outcomes pertain to Path 1. In the DCS-SOMP case, $q_{az}$ and $q_{el}$ are both set to 18, whereas in the adaptive DCS-SOMP scenario, $q_{az}$ and $q_{el}$ are both set to 6.

**Figure 6:**
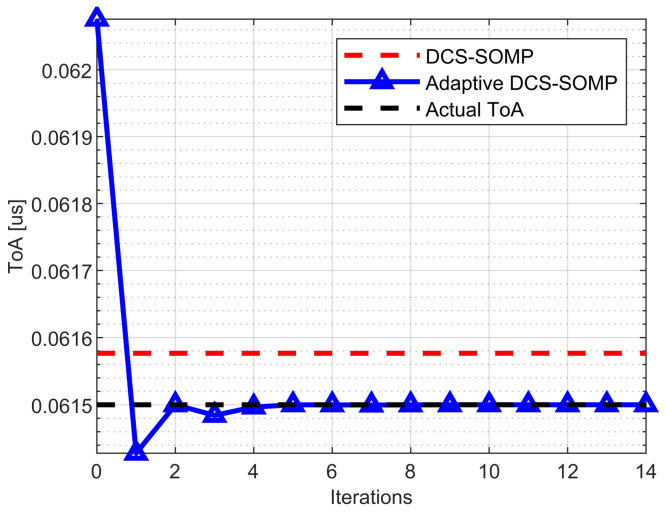
Comparison of ToA estimation results using DCS-SOMP and adaptive DCS-SOMP. The outcomes pertain to Path 1. In the DCS-SOMP case, $q_{az}$ and $q_{el}$ are both set to 18, whereas in the adaptive DCS-SOMP case, $q_{az}$ and $q_{el}$ are both set to 6.

Figure 4a, Figure 5 and Figure 6 display the comparison between DCS-SOMP and adaptive DCS-SOMP for 2D-AoD, 2D-AoA, and ToA. The results correspond to a random run for Path 1. It’s evident that, for all cases, the adaptive DCS-SOMP significantly enhances the estimation of the respective angle as early as the second iteration, gradually converging towards the actual value with each subsequent iteration.

We analyzed the complexity of the methods in terms of execution time and number of mathematical operations. Following the analysis provided in [18,19], the complexity of the SOMP algorithm is $O(LN_{t}q_{c})$, where $q_{c}=q_{az}q_{el}$ denotes all possible combinations for the candidates for azimuth ($q_{az}$) and elevation angles ($q_{el}$). In the DCS-SOMP algorithm, we perform the Kronecker product (tensor product for matrices) to estimate 2D-AoD and 2D-AoA parameters, resulting in $O(LN_{t}{\left(q_{c}\right)}^{2})$. In our adaptive DCS-SOMP approach, we fix the number of candidates and we update the values for the candidates at each iteration. Thus, the complexity of the adaptive DCS-SOMP is $O(LN_{t}q_{c}q_{it})$, where $q_{it}$ is the number of iterations. The main mathematical operations are due to (7) and (8). We determine the total number of mathematical operations, $q_{op}$, as follows:

(24) $$q_{op}=\left(q_{op}^{w}+q_{op}^{h}\right)\left(q_{it}L+1\right),$$

where $q_{op}^{w}$ is the number of mathematical operations to construct the sensing matrix, (7), determined as follows:

(25) $$q_{op}^{w}=N\left(q_{c}M_{t}N_{t}+q_{c}\left(M_{t}N_{t}-1\right)+M_{t}N_{t}{\left(q_{c}\right)}^{2}\right),$$

and $q_{op}^{h}$ is the number of mathematical operations to determine the max correlation, (8), determined as follows:

(26) $$q_{op}^{h}=\left(2N_{t}M_{t}\right)N{\left(q_{c}\right)}^{2}.$$

Table 3 presents the execution times and the number of mathematical operations for each method. For the adaptive DCS-SOMP method, we utilized 14 iterations. These values represent the total duration required by the method to estimate all five parameters for both paths. Our observations indicate that the adaptive method is notably faster and involves fewer mathematical operations compared to the traditional method.

We used the Root Mean Square Error (RMSE) as the Key Performance Indicator (KPI) for our estimates. The RMSE ε is calculated as follows:

(27) $$\varepsilon =\sqrt{\frac{1}{Q}\sum_{i=1}^{Q}{\left|p_{i}-{\hat{p}}_{i}\right|}^{2}},$$

where *Q* is the number of runs, $p_{i}$ is the actual value of the chosen parameter at the *i*-th run, and ${\hat{p}}_{i}$ is the estimated value of the chosen parameter at the *i*-th run.

Table 4 presents the RMSE for each parameter estimated, where $\varepsilon_{\phi^{az}}$, $\varepsilon_{\phi^{el}}$, $\varepsilon_{\theta^{az}}$, $\varepsilon_{\theta^{el}}$, and $\varepsilon_{\tau }$ represent the RMSE for azimuth AoD, elevation AoD, azimuth AoA, elevation AoA, and ToA, respectively. The RMSE was determined from 100 runs of each method, considering the average across all paths. The adaptive method exhibits higher precision compared to the non-adaptive method for all analyzed parameters.

## 6. Conclusions

In this work, we addressed the problem of low performance presented by traditional DCS-SOMP approaches, which present convergence and precision to estimate parameters only at the cost of low performance. To achieve this, we proposed an adaptive DCS-SOMP method that dynamically calculates the sensing matrix, presenting high performance in precisely estimating localization parameters while keeping the algorithm simple and with fast convergence.

This modification transformed the DCS-SOMP method from solely a coarse estimator to a singular tool for precise parameter estimation. Even in a 3D environment with five parameters to be estimated and multiple paths to be detected, the adaptive DCS-SOMP method exhibited substantial improvements in both accuracy and speed compared to the DCS-SOMP approach. Further enhancements to the adaptive DCS-SOMP could be explored, such as refining the way the residue was updated, potentially enabling improved separation of paths. Furthermore, we plan to explore alternative antenna arrays, including 2D configurations, to enhance beam-forming capabilities in mmWave scenarios within 5G and B5G networks.

## Figures and Tables

**Table 1:** Related work overview.

Article	Method	Array	ToA	2D-AoD	2D-AoA
[5]	DCS-SOMP	ULA	✔	×	×
[8]	MUSIC	ULA	✔	×	✔
[10]	SBT	ULA	×	×	×
[11]	MUSIC	URA	×	✔	✔
[12]	SSFEAL	UCA	×	×	✔
**Our Proposal**	Adaptive DCS-SOMP	UCA	✔	✔	✔

**Table 2:** Actual Values Used in Simulations.

Path	$\phi^{az}$ (rad)	$\phi^{el}$ (rad)	$\theta^{az}$ (rad)	$\theta^{el}$ (rad)	τ (us)
1	1.33	0.45	3.50	0.90	0.0615
2	2.80	1.15	5.20	1.45	0.0767

**Table 3:** Comparison between DCS-SOMP and Adaptive DCS-SOMP.

Method	Time (s)	Number of Mathematical Operations
DCS-SOMP	84.63	4.0393 × 10^9^
Adaptive DCS-SOMP	0.88	1.4697 × 10^9^

**Table 4:** Comparison of RMSE between DCS-SOMP and adaptive DCS-SOMP.

Method	$\varepsilon_{\phi^{az}}$ (rad)	$\varepsilon_{\phi^{el}}$ (rad)	$\varepsilon_{\theta^{az}}$ (rad)	$\varepsilon_{\theta^{el}}$ (rad)	$\varepsilon_{\tau }$ (us)
DCS-SOMP	0.1334	0.0454	0.1087	0.2851	0.0109
Adaptive DCS-SOMP	0.0017	0.0005	0.0089	0.0256	0.0001

## Data Availability

Data is contained within the article.
